# A Review of Femtosecond Laser Processing for Sapphire

**DOI:** 10.3390/ma19010206

**Published:** 2026-01-05

**Authors:** Chengxian Liang, Jiecai Feng, Hongfei Liu, Yanning Sun, Yilian Zhang, Yingzhong Tian

**Affiliations:** 1 School of Mechatronic Engineering and Automation, Shanghai University, Shanghai 200444, China; 25722184@shu.edu.cn (C.L.); sunyanning@shu.edu.cn (Y.S.); zhangyilian@shu.edu.cn (Y.Z.); troytian@shu.edu.cn (Y.T.); 2 The Aviation Services Research Centre, The Hong Kong Polytechnic University, Hung Hom, Kowloon, Hong Kong; hongfei.liu@polyu.edu.hk

**Keywords:** sapphire, femtosecond, laser processing, micro/nano structure fabrication, surface modification, direct writing

## Abstract

Sapphire (α-Al_2_O_3_) has been widely used in high-power lasers, optical windows, semiconductor substrates, radomes, and other applications due to its exceptional optical properties, high hardness, excellent chemical stability, and thermal resistance. However, machining sapphire poses significant challenges because of the material’s high hardness and brittleness. Traditional mechanical and chemical–mechanical machine methods often fail to meet the processing requirements for micro and nanoscale structures. Recently, the use of femtosecond lasers—with ultra-short pulses and extremely high peak power—has allowed for the precise machining of sapphire with minimal thermal damage, a method akin to cold processing. Femtosecond laser processing offers significant advantages in fabricating three-dimensional micro- and nanoscale structures, surface and internal modification, optical waveguide writing, grating fabrication and dissimilar materials welding. Thus, this paper systematically reviewed the research progress in femtosecond laser processing of sapphire, covering technical approaches such as ablation, hybrid processing and direct writing micro- and nanoscale fabrication. The capability of femtosecond laser processing to modulate sapphire’s optical properties, wettability and mechanical and chemical characteristics were discussed in detail. The current challenges related to efficiency, cost, process standardization and outlines future development directions, including high-power lasers, parallel processing, AI optimization and multifunctional integration were also analyzed.

## 1. Introduction

Sapphire, also known as corundum, is primarily composed of Al_2_O_3_. Trace elements such as iron and titanium give it colors ranging from sky blue to pale blue. Sapphire exhibits excellent optical properties, mechanical strength, and chemical stability [1,2], making it widely used in high-power laser devices [3], optical windows for extreme temperature and pressure environments [4], and as a substrate material for semiconductors and large-scale integrated circuits [5,6]. Additionally, sapphire can be fabricated into critical optical components such as lenses, prisms, and mirrors. In mechanical engineering, sapphire is predominantly used for precision instrument housings and precision mechanical bearings [7]. Within electronics, sapphire serves as a material for constructing detection instruments like gravitational wave detectors [8]. In industry applications, sapphire finds extensive use in key equipment including missile fairings, optical windows, infrared devices [9,10], and submarine periscopes [11]. Its high strength, superior light transmission, and extreme environment resistance significantly enhance equipment performance. In medicine, sapphire-based laser therapy provides a painless, non-invasive medical treatment. By generating coherent, monochromatic light that ensures precise tissue targeting with minimal thermal spread, this technology achieves therapeutic effects through controlled photothermal interactions without the risk of adverse drug reactions [12].

**Figure 1:**
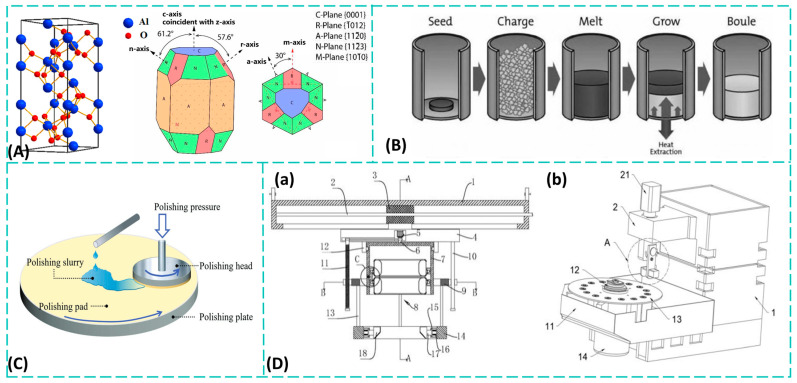
(**A**) Three-dimensional schematic diagram of sapphire crystal structure and crystal planes [5,14], The arrows in the figure indicate crystallographic directions, the numbers show the angles between specific lattice planes or axes; (**B**) Schematic diagram of sapphire crystal growth via the thermal exchange method [15]; (**C**) Schematic diagram of sapphire processing by chemical–mechanical polishing [16]; (**D**) Prototype diagrams of sapphire grinding apparatus (**a**) and polishing apparatus (**b**).

Sapphire is a crystal with a hexagonal lattice structure, featuring five distinct crystal faces: C-face, A-face, M-face, N-face, and R-face [13]. Figure 1A illustrates a three-dimensional schematic of the sapphire crystal structure and its crystal faces [5,14]. The optical axis of sapphire is perpendicular to its C-plane. Its physical and chemical properties exhibit pronounced anisotropy along this axis and the directions parallel to it. When oriented along the C-axis, sapphire shows no birefringence in that direction. The C-plane has the highest hardness and optimal mechanical symmetry, making designs that utilize the C-axis direction a common choice for optical components. 

In addition to its outstanding mechanical properties, sapphire possesses exceptional thermal and chemical stability. Its broad-spectrum high transmittance is a key optical advantage, with a transmission range spanning from ultraviolet to mid-infrared wavelengths. Typically, its transmittance exceeds 80% across the 0.4–2.5 μm range. The refractive index is approximately 1.76 and remains stable with temperature variation. Mechanically, it exhibits exceptional hardness, with a Mohs hardness of 9 and a Vickers hardness of approximately 2200 kg/mm^2^, contributing to its remarkable wear resistance. Its tensile strength is approximately 400 MPa, flexural strength around 895 MPa, and compressive strength can reach 2 GPa. Despite its outstanding hardness and strength, sapphire has a Poisson’s ratio of only 0.28, classifying it as a typical brittle material with poor impact resistance. Regarding thermal properties, sapphire has a high melting point of 2045 °C, with a thermal conductivity of 30.3 W/(m·K) perpendicular to the C-axis and 32.5 W/(m·K) parallel to it. Both values exceed those of conventional optical materials like quartz and most dielectrics, making sapphire suitable for high-temperature applications. It maintains excellent mechanical and optical properties under elevated temperatures or rapid thermal cycling, demonstrating exceptional thermal stability and superior thermal conductivity. Electrically, sapphire exhibits a resistivity of 1 × 10^11^ Ω·cm, with a dielectric constant of 11.5 along the C-axis. It is an excellent electrical insulator that maintains good insulation properties even at high temperatures. Its dielectric strength is approximately 480 kV/mm, featuring a high breakdown voltage suitable for high-voltage applications. Sapphire is also frequently used as an epitaxial substrate for semiconductor materials such as gallium nitride and silicon. Regarding chemical properties, sapphire exhibits exceptional chemical stability. At room temperature, it resists corrosion by strong acids and alkalis and does not undergo redox reactions under normal conditions. However, at elevated temperatures, sapphire reacts to some extent with hydrofluoric acid, phosphoric acid, and potassium hydroxide, and may interact with reactive metals such as potassium and sodium.

Currently, synthetic sapphire technology has matured significantly, with production processes encompassing solid-phase, liquid-phase, and gas-phase methods [15,17,18]. Among these, liquid-phase synthesis has gained widespread industrial adoption due to its ability to rapidly produce high-quality, large-size sapphire single crystals. Mainstream liquid-phase growth processes include the crucible-lowering method, heat-exchange method [19], Czochralski method [20], and bubble-growth method [21]. Figure 1B illustrates the crystal growth process of the heat-exchange method. Among these, the Czochralski method is one of the most commonly used techniques for preparing large-diameter, high-quality sapphire single crystals via the melt-growth approach. This method begins by heating the crystal growth raw material in a crucible to a molten state. By precisely controlling the temperature distribution within the growth furnace, a supercooled region forms at the top of the melt. Subsequently, the seed crystal at the tip of the seed rod is brought into contact with the melt, causing localized melting on its surface. As the seed rod is lifted and rotated, the supercooled melt begins to crystallize on the seed crystal surface. With the continued lifting and rotation of the rod, ordered atomic or molecular arrangements continuously occur at the solid–liquid interface, promoting the gradual solidification and elongation of the melt into a complete single crystal. Characteristics of the pulling method include [22,23]: (1) Eliminating inherited dislocations through seed rod necking enhances crystal integrity. (2) Rapid crystal growth enables preparation of single crystals with different orientations using seeds. (3) Direct testing and observation during growth facilitates precise control of growth conditions.

As related technologies advance toward high performance, miniaturization, and integration, demands for the processing quality and precision of sapphire components are becoming increasingly stringent. However, sapphire’s high hardness and brittleness make it a notoriously difficult material to machine. Traditional mechanical processing methods primarily include ultra-precision cutting using diamond turning tools [24], grinding and polishing with ultra-fine abrasive belts, and ultra-precision grinding with diamond micro-powder grinding wheels. These techniques encompass the core processes of cutting, shaping, and polishing, which are essential for transforming raw sapphire into functional devices with precise geometries and surface finishes [25]. As illustrated in Figure 1D, specialized grinding and polishing apparatus are fundamental to this fabrication workflow. The production of many industrial sapphire components—from complex shapes like radomes [26] to items requiring ultra-smooth surfaces such as high-end watch crystals [27]—relies heavily on these established machining methods to achieve the necessary dimensional accuracy and surface quality. While suitable for large-scale optical elements, these methods struggle to meet the machining demands of micro- and nano-scale optical microstructures. Chemical–mechanical polishing (CMP) technology combines mechanical grinding and chemical etching mechanisms. Through the synergistic action of ultra-fine particle abrasion and chemical reactions within the polishing solution, it achieves low-roughness, damage-free, flat, and smooth surfaces on sapphire processing samples [28,29,30]. Its working principle is illustrated in Figure 1C [16]. By introducing chemical reactions to traditional mechanical processing, this technique significantly enhances processing efficiency and improves surface roughness. However, it remains primarily applicable to planar sapphire samples and is challenging to use for fabricating sapphire devices with micro/nano structures.

**Figure 2:**
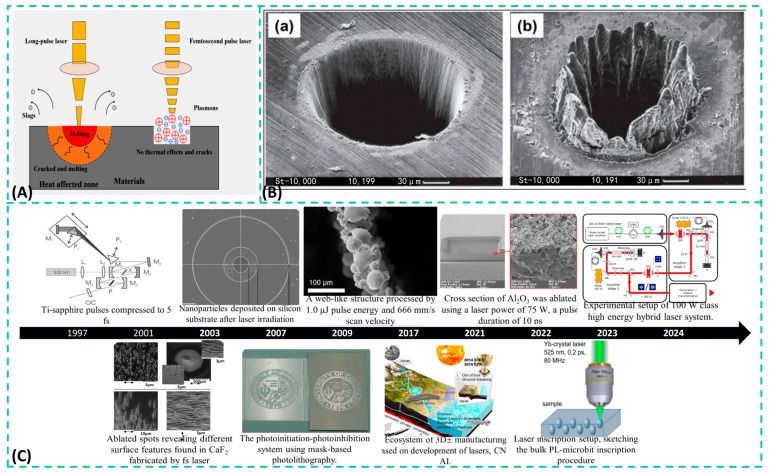
(**A**) Interaction processes between long-pulse laser (**left**)/femtosecond laser (**right**) and materials [32], In long-pulse laser processing, thermal energy continuously diffuses into the material and its surroundings; in femtosecond laser processing, the material becomes highly ionized and is ejected from the processing area in the form of plasma; (**B**) Microporous structures prepared by femtosecond laser (**a**) and picosecond laser (**b**) ablation of steel [31]; (**C**) A brief history chart of major breakthroughs in ultrafast lasers and manufacturing processes from 1997 to 2024 [31].

Compared to traditional processing methods, pulsed laser processing, an advanced manufacturing technique that has gradually evolved over the past few decades, has significantly outperformed conventional approaches in machining brittle and hard materials such as sapphire. This success is due to continuous improvements in laser performance, stability, and efficiency. Figure 2C illustrates the brief history of ultrafast lasers and manufacturing processes [31]. This timeline outlines the evolution of ultrafast laser technology and precision manufacturing since 1997. It progresses from basic femtosecond pulse compression and surface modification to advanced applications like 3D micro/nano fabrication, hybrid laser-lithography systems, and integrated digital manufacturing platforms, highlighting the field’s continuous advancement towards greater complexity and integration. Pulsed lasers can be primarily categorized into long-pulse lasers, short-pulse lasers, and ultrashort-pulse lasers based on their pulse width. Long-pulse lasers deliver millisecond- to microsecond-duration pulses onto the workpiece surface. Due to their extended pulse durations, long-pulse lasers primarily perform thermal processing on materials. During sapphire microstructure fabrication, thermal stresses can easily cause defects such as chipping, cracking, or fragmentation around the microstructures. Therefore, long-pulse lasers are unsuitable for processing sapphire. In contrast, short-pulse lasers, with durations in the nanosecond range, and ultra-short-pulse lasers, with durations in the picosecond or femtosecond range, have significantly shorter pulse widths. Their brief interaction times enable the delivery of higher energy to the material surface in a shorter period, thereby minimizing thermal effects on the material. Figure 2A [32] illustrates the interaction process between long-pulse/femtosecond lasers and materials, while Figure 2B shows microporous structures prepared by femtosecond and picosecond laser ablation of steel [33]. It is evident that femtosecond lasers exhibit superior processing precision and cause less surface damage compared to other ultrashort pulse lasers.

In recent years, ultrafast laser micro/nano fabrication has emerged as one of the frontier areas in the development of processing technologies [34,35,36]. The significant reduction in pulse width and substantial increase in peak power have granted femtosecond lasers a leading edge in micro-processing and manufacturing. Moreover, the interaction between femtosecond lasers and materials exhibits high precision and accuracy, fully meeting the requirements of nanoscale processing [37,38,39]. Featuring an extremely short temporal scale (10^−15^ s) and high energy density (>10^14^ W/cm^2^), femtosecond lasers can be precisely focused to the nanoscale (10^−9^ m) [40,41,42]. It should be noted that achieving such a small focal spot typically requires overcoming the classical optical diffraction limit. This can be realized through advanced techniques such as near-field effects (using scanning near-field optical microscopy probes), the utilization of solid immersion lenses, or the design of metasurface-based super-oscillatory lenses. Furthermore, methods like stimulated emission depletion (STED) inspired laser processing can effectively narrow the machining region to the nanometer level. These approaches, combined with the ultrashort pulse characteristics of femtosecond lasers that minimize thermal diffusion, enable truly nanoscale, high-precision material processing.

During femtosecond laser micromachining, internal heat generation and diffusion remain negligible. Within the ultrashort pulse duration (typically tens to hundreds of femtoseconds), photons are primarily absorbed by free and bound electrons, elevating them to a high-energy non-equilibrium state. The critical “cold” machining characteristic arises from the distinct time-scale separation between electron excitation and the subsequent electron–phonon coupling process. The absorbed energy is initially confined within the electron subsystem, leading to a rapid increase in electron temperature. The transfer of this energy to the lattice (phonons) through electron–phonon collisions—a process governing the eventual thermalization and potential phase change of the material—occurs on a significantly longer picosecond timescale. Since the laser pulse ends before this energy transfer is complete, the direct thermal diffusion and conventional melting caused by lattice heating are substantially suppressed [43,44]. This mechanism enables precise, localized ablation with minimal heat-affected zones.

This paper reviews recent advances in femtosecond laser processing of sapphire. It begins by outlining three methods for fabricating three-dimensional micro- and nanostructures on sapphire surfaces: femtosecond laser ablation, hybrid femtosecond laser processing, and direct femtosecond laser micromachining. Next, it examines the effects of femtosecond lasers on sapphire’s optical properties, damage threshold, hydrophilicity/hydrophobicity, and other characteristics. Finally, the paper discusses common processing techniques such as writing waveguides, fabricating gratings, and welding metals onto sapphire using femtosecond lasers. It concludes by summarizing the challenges and corresponding strategies across various application areas in this field.

## 2. Fabrication of Surface Three-Dimensional Micro/Nano Structures

Femtosecond laser-based three-dimensional micro/nano-structure fabrication on sapphire surfaces is an advanced processing technology that relies on the interaction between ultrafast lasers and matter. Utilizing extremely short pulses and exceptionally high peak power, this technique employs mechanisms such as nonlinear multiphoton absorption to achieve high-precision, controllable three-dimensional structuring within either or on the surface of sapphire, a material characterized by high hardness, chemical stability, and a wide bandgap. During processing, the laser focus is scanned across the sapphire interior or surface using a precision three-dimensional motion control system. By adjusting laser parameters and focal position, the sapphire surface can be precisely modified.

### 2.1. Femtosecond Laser Ablation

Femtosecond laser ablation processing of sapphire finds extensive application in three-dimensional micro/nano structure fabrication. During ablation, sapphire directly transitions from solid to gas or plasma state, reducing liquid material accumulation at the bottom of ablation holes. This facilitates direct laser energy interaction with the pole bottom, enabling greater ablation depth, which is closely related to the interaction time between laser and material. Tian et al. [45] investigated the ablation behavior of sapphire under 780 nm femtosecond laser irradiation. They observed that the ablation diameter increases with energy density, though the growth rate gradually slows down. This phenomenon is closely related to the normal distribution of spot energy. Li et al. [46] further compared the effects of 400 nm and 800 nm femtosecond lasers, finding that shorter pulses produced finer ablation pits. Under 800 nm, 45 fs conditions, the ablation threshold for sapphire was measured at approximately 4.5 J/cm^2^. Kudrius et al. [47] indicated that structural changes induced by femtosecond lasers on sapphire surfaces are associated with oxygen vacancies. High-temperature heat treatment can eliminate these defects, thereby enhancing LED device performance. The most critical and valuable aspect of these studies lies in the systematic quantification of the relationship between ablation thresholds and pulse width/wavelength, providing a basis for defining the “cold processing” window. However, thermal accumulation-induced microdamage remains a challenge during femtosecond laser ablation, particularly at high repetition rates. To mitigate this issue, future approaches may integrate real-time monitoring techniques such as pump-probe imaging to dynamically adjust laser parameters, thereby suppressing thermal effects and minimizing microdamage.

**Figure 3:**
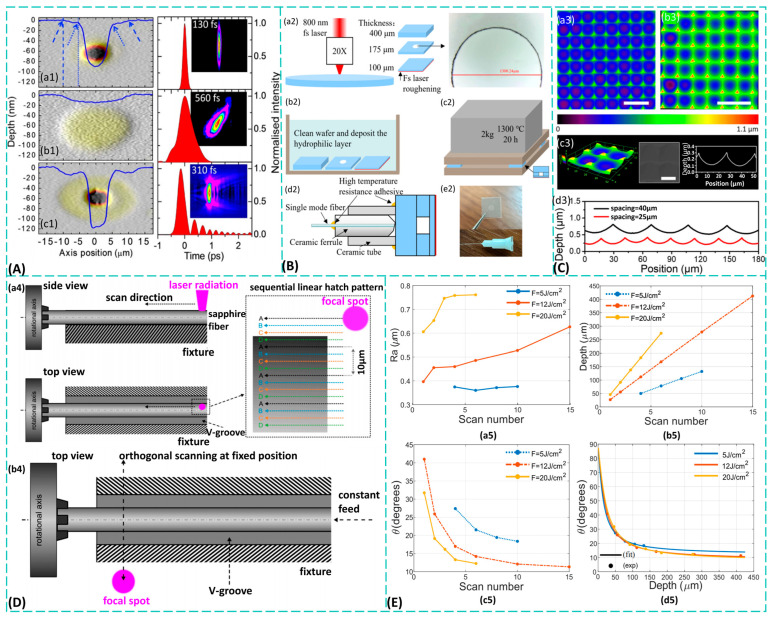
(**A**) False color topography images (left column) and crater depth cross sections of laser irradiated regions with (**a1**) single TL, (**b1**) ST, and (**c1**) TOD-shaped pulses. The experimentally measured temporal pulse intensity profiles are shown in the right column [50]. The insets show the PG-FROG traces with a 30 nm spectral window (vertical axis) and 6 ps for the temporal window (horizontal axis). Irradiations were performed at different fluences: (**a1**) 8.3 J/cm^2^; (**b1**) 12.6 J/cm^2^; (**c1**) 14.8 J/cm^2^; (**B**) Sensor fabrication process [57]: (**a2**) femtosecond laser micromachining; (**b2**) sapphire wafer cleaning; (**c2**) direct bonding; (**d2**) sensor encapsulation; (**e2**) physical photograph of the sensor; (**C**) Characterization of the concave microlens array on the sapphire surface [59]: (**a3**,**b3**) Top-view CLSM images of microlens arrays with 25 and 40 μm pitch, respectively; (**c3**) Detailed 3D view, SEM image, and corresponding cross-sectional profile of the concave microlens in (**a3**); (**d3**) Cross-sectional profiles of the concave microlenses in (**a3**) and (**b3**). Scale bar: (**a3**) 50 μm, (**b3**) 80 μm, and (**c3**) 20 μm; (**D**) (**a4**) Radial reduction of sapphire rods achieved via femtosecond laser using axial scanning combined with progressive workpiece rotation; (**b4**) Diameter reduction of 20 cm sapphire rods via femtosecond laser using orthogonal scanning [64]; (**E**) Trends of different parameters in sapphire with scanning iterations: (**a5**) roughness, (**b5**) depth, (**c5**) cone angle, (**d5**) variation in cone angle with depth [65].

To effectively control the sapphire ablation process, numerous parameters of the femtosecond laser play a crucial role in ablation. Shaheen et al. [48] systematically investigated the effects of laser energy density, repetition rate, and pulse number on ablation rate, finding that high energy density combined with high repetition rate enhances plasma effects and improves ablation efficiency. Chang et al. [49] proposed using Yb femtosecond lasers for sapphire surface patterning, discovering that low energy density combined with rapid scanning facilitates the formation of pit-free microstructures. To demonstrate the controllability of ablation mechanisms via pulse shape, Hernandez-Rueda et al. [50] experimentally analyzed pit morphology evolution under single TL, ST, and TOD pulses. They plotted pulse shapes alongside ablation pit morphology images and depth cross-sections, as shown in Figure 3A. Achieving desired processing effects by altering laser parameters typically requires only new data templates during machining. However, frequent parameter changes necessitate preparing numerous distinct laser heads, causing costs to scale proportionally with processing frequency. In contrast, pulse shaping emerges as a promising technology that enables active regulation of ablation mechanisms. By employing simplified pulse control modules combined with real-time waveform optimization through artificial intelligence, it enhances processing flexibility and consistency while reducing costs.

In the sapphire femtosecond laser ablation process, precise control of parameters is essential for achieving high-quality the extremely brief duration of the femtosecond pulse, which ensures that energy deposition occurs before thermal diffusion can take place. This allows for precise material removal without thermal damage through nonlinear absorption. The laser wavelength influences the efficiency of nonlinear absorption; shorter wavelengths generally lower the ablation threshold and enhance precision. Pulse energy and fluence must be carefully controlled to remain just above the material’s ablation threshold, insufficient energy prevents effective processing, while excessive energy causes thermal effects and damage. This control is critical for maintaining processing quality. The scanning speed determines the pulse overlap rate, which affects processing uniformity and thermal management. The beam’s focusing state directly governs the ultimate precision and contour of the processing. Together, these parameters form a precise, synergistic system. Any mismatch in any component will directly impact the final product’s processing efficiency, surface quality, and geometric accuracy.

Femtosecond laser ablation technology is predominantly applied in the fabrication of complex micro- and nano-structures in sapphire, such as micro-holes, capillaries, and grooves. Cai et al. [51] demonstrated bidirectional drilling using femtosecond lasers, successfully producing crack-free microthrough holes in sapphire. By adjusting the defocus distance and pulse energy, they suppressed the formation of anomalous regions. Similarly, Zhang et al. [52] proposed a strategy of linearly increasing energy with depth, enhancing the roundness and depth uniformity of sapphire capillaries. Extending these approaches, Liu et al. [53] in combined plasma-assisted ablation with direct ablation to achieve crack-free microgrooves with a high aspect ratio of 10:1. In the fabrication of these micro- and nanostructures, most methods rely on the coordination of multiple mechanisms and dynamic parameter control to achieve high-quality production of complex sapphire structures. The energy-as-a-function-of-depth compensation strategy holds significant engineering value, particularly for deep-hole and deep-groove processing. However, most current methods still face challenges related to low efficiency and high cost. Future research could explore multi-beam parallel processing or laser-ultrasonic hybrid techniques to improve processing efficiency and consistency.

Femtosecond laser ablation offers distinct advantages for sapphire processing, particularly its near-“cold processing” characteristics. This allows for high-precision machining with minimal thermal damage. The extremely short pulse duration of femtosecond lasers prevents energy from diffusing as heat within the processed area. Instead, material is removed through nonlinear absorption and direct vaporization, effectively avoiding thermal damage issues commonly encountered when processing hard, brittle materials like sapphire, such as microcracks and remelted layers [54]. This process produces sharp edges and smooth surfaces, while adjusting laser parameters enables varied ablation outcomes to improve machining precision. However, the technology has significant drawbacks: high cost and low efficiency. The system is expensive, and its relatively low average power generally leads to slower material removal rates and processing speeds compared to traditional long-pulse lasers or mechanical methods. Consequently, it is economically unfeasible for mass production of large-scale macro-components. Additionally, the stringent requirements for precise parameter control and the high technical expertise needed during processing present challenges for process optimization and widespread adoption.

### 2.2. Femtosecond Laser Hybrid Processing

Femtosecond laser hybrid processing technology aims to overcome the limitations of single-laser processing in terms of efficiency, quality, and material adaptability by combining femtosecond lasers with other physical or chemical processes to achieve synergistic effects. Building on the preceding discussion of single ablation techniques, the core of hybrid processing lies in leveraging the precision and cold-processing characteristics of femtosecond lasers as a guiding or driving source, while utilizing the advantages of auxiliary methods to enhance efficiency. Among these, femtosecond laser etching of sapphire represents an advanced manufacturing technique that employs ultra-short, ultra-high-intensity laser pulses for precise During this process, energy is deposited into the electronic system before thermal diffusion occurs on the picosecond scale. Through mechanisms such as multiphoton absorption, the material undergoes direct plasma formation and removal, thereby minimizing thermal damage such as melting and microcracking. Etching techniques are primarily categorized into dry etching and wet etching.

Specifically, the fundamental principle of the composite technology combining dry etching with femtosecond laser processing [55] involves rapidly forming chemically modified zones within the material using low-energy femtosecond laser pulses. The deposition of pulse energy endows these modified zones with heightened physicochemical reactivity, thereby achieving higher etching rates. Building upon this, Zhang et al. [56] and Yi et al. [57] employed femtosecond lasers for dry etching and surface roughening of sapphire wafers, developing an all-sapphire-based optical fiber pressure sensor. The fabrication process is illustrated in Figure 3B. This sensor exhibits three key characteristics: ultra-high pressure tolerance, high-temperature resistance, and intrinsic safety. Dry etching-assisted techniques ingeniously circumvent the low direct ablation efficiency of femtosecond lasers. By adopting a “laser modification + selective etching” approach, they transform processing challenges into problems of selective material activation. Zhang and Yi et al. [56,57] demonstrated how to translate this micro/nanofabrication technology from the laboratory to practical applications. They successfully integrated multiple processes, through-hole etching, surface roughening, and wafer bonding, to develop high-performance sensors for extreme environments. This work highlights the significant potential of femtosecond laser hybrid processing for achieving multifunctional device integration. Their research established a universal modification followed by assisted removal process. However, a major challenge remains the inherent requirements of dry etching, which involves costly vacuum equipment and specific reactive gases. The key task is to explore more diverse, low-cost auxiliary methods, such as developing specialized chemical wet etchants for laser-modified regions or investigating the feasibility of plasma-assisted etching in ambient air to reduce process barriers.

On the other hand, wet etching auxiliary techniques also demonstrate unique advantages. This process involves immersing the wafer in a specific chemical solution, where the solution reacts purely chemically with the exposed material, dissolving and removing it. The chemical reactants in the solution selectively interact with the target material, forming water-soluble products. This enables us to achieve high-speed etching of one material while significantly slowing the etching rate of another by selecting different chemical solutions, thereby effectively protecting underlying or masked materials. Sun et al. [58] proposed an efficient self-modulated femtosecond laser hybrid technique. By combining back-side laser modification with wet etching, they overcame the depth limitations of traditional LIPSS and fabricated periodic nanostructures on sapphire. These structures achieved aspect ratios exceeding 55, meaning their depth was more than 55 times greater than their lateral period. Similarly, Cao et al. [59] employed an innovative process integrating femtosecond laser holographic processing with wet etching to achieve rapid, maskless fabrication of concave sapphire microlens arrays. Imaging performance was characterized using a test system, with results shown in Figure 3C, demonstrating significantly enhanced processing efficiency. Sun et al. [58] pushed hybrid processing capabilities to new heights, achieving high aspect ratio nanostructures unattainable by conventional methods. Their “self-modulation” mechanism is particularly ingenious, potentially involving self-organization phenomena arising from laser-matter interactions, establishing a new paradigm for fabricating periodic subwavelength structures. Cao et al. [59] ingeniously combined the holographic parallel processing capability of femtosecond lasers with the high efficiency of wet etching, resolving the trade-off between efficiency and precision in fabricating complex structures like microlens arrays. Their findings demonstrated that wet etching can efficiently and precisely remove femtosecond laser-induced modified regions, enabling cross-scale manufacturing from the nanometer to micrometer scale.

Extending this discussion, the mechanism of femtosecond laser interaction with materials prior to wet etching plays a critical role. When etching sapphire with femtosecond lasers, the ultra-short pulses initially induce nonlinear interactions within the material. By precisely controlling the energy density, selective scanning modifies the crystal lattice structure, creating a chemically more reactive altered layer without directly removing bulk material. Subsequently, the laser-treated sapphire is immersed in a specific chemical etching solution. The etching rate of the laser-modified regions is significantly higher than that of the unmodified areas, enabling selective dissolution and the high-precision formation of pre-designed patterns with high aspect ratios. Liu et al. [60] developed a combined femtosecond laser deep scribing technique with etching processes to fabricate high-quality microstructures on sapphire surfaces with aspect ratios up to 80:1. Jiang et al. [61] proposed a hybrid process integrating femtosecond laser pulse patterns with wet chemical etching, achieving efficient microhole fabrication at 4000 holes per second. Xie et al. [62] employed femtosecond laser-assisted chemical etching to form both pyramidal and tetrahedral micro/nano structures on sapphire surfaces, demonstrating their potential applications across diverse fields. Liu et al. [60] introduced an insightful approach using a silica sacrificial layer, effectively isolating surface damage from the internal modification zone to ensure controllability during subsequent etching. This “protective” mindset is crucial for processing complex structures. Xie et al. [62] demonstrated that active control over final structural morphology can be achieved by adjusting laser and chemical parameters. Hybrid processing is not a simple sequential execution but a complex system of parameter coupling. This approach unifies high aspect ratio, high efficiency, and controllable complex topography. However, both research streams face shared challenges. Extensive experiments have revealed a contradiction between the isotropic nature of the etching process and the anisotropic properties of sapphire crystals, which can lead to deviations in structural morphology from the intended design. By thoroughly investigating the corrosion kinetics of different crystal planes in specific etching solutions and designing laser scanning paths based on crystal orientation, post-etching structures can be made to better conform to design requirements to a certain extent. Furthermore, a common challenge remains: ensuring structural consistency and uniformity during high-volume, large-area processing. This issue can be addressed by developing online monitoring technologies that provide real-time feedback on etching rates and topographical changes. When combined with machine learning algorithms that dynamically adjust laser parameters or etching conditions, adaptive manufacturing can be achieved.

Beyond integration with etching techniques, femtosecond lasers also demonstrate significant potential when combined with traditional machining methods. Katehira et al. [63] employed a hybrid manufacturing process integrating femtosecond laser processing with diamond tool micro-milling. By synergizing the strengths of both techniques, highly efficient and precise machining of sapphire was achieved. The femtosecond laser first modifies the material, overcoming sapphire’s inherent high hardness and brittleness. This modification enables subsequent mechanical machining to proceed in a near-plastic manner, avoiding issues such as tool wear and edge chipping. This softening + mechanical precision carving approach pioneers a novel pathway for machining complex three-dimensional structures in hard and brittle materials, delivering both high efficiency and superior surface quality. However, a critical challenge remains in precisely controlling the depth and uniformity of the modified layer. This control is essential to ensure complete removal of the modified layer during machining without damaging the underlying pristine material. Potential solutions include developing quantitative models that correlate laser modification parameters with variations in material hardness and brittleness, as well as creating in-situ detection technologies to verify real-time removal of the modified layer.

### 2.3. Femtosecond Laser Direct Writing for Micro/Nano Manufacturing

Femtosecond laser direct writing is a single-step, precision manufacturing technique for micro/nano fabrication. It relies on nonlinear absorption induced by ultrashort pulse durations and high peak power, enabling precise material modification with minimal thermal damage. This direct-write process creates high-resolution features by scanning a tightly focused beam in three dimensions without the need for masks or post-processing. It is particularly valuable for fabricating complex sapphire components—such as optical windows, sensors, and microchannels—used in microelectronics, integrated photonics, and microfluidic chips. Ng et al. [66] employed femtosecond laser direct writing to fabricate diverse three-dimensional surface topographies on single-crystal sapphire wafers. They quantified the relationship between laser processing parameters and the resulting three-dimensional functional surface morphology, enabling precise fabrication of three-dimensional surface structures on single-crystal sapphire without iterative trial-and-error. Similarly focused on complex structure processing, Kefer et al. [64] demonstrated two femtosecond laser direct writing microfabrication techniques specifically tailored for processing rotationally symmetric sapphire workpieces. The final processed workpieces achieved an astonishing aspect ratio of 2200, with the two laser scanning methods illustrated in Figure 3D. The significance of Ng et al.’s [66] research lies in shifting femtosecond laser direct microfabrication from an “art” or “experience-based” approach toward a “scientific” one. Their established parameter-morphology relationship model substantially reduces trial-and-error costs in process development, marking a crucial step toward intelligent processing. Kefer et al. [64] demonstrated the ultimate capability of femtosecond lasers in processing specific geometric shapes (rotationally symmetric workpieces). The achieved aspect ratio demonstrates the immense potential of femtosecond lasers in micro- and nanoscale machining, providing effective solutions for manufacturing micro-electro-mechanical system (MEMS) components such as miniature bearings and probes. Both micro-machining techniques enable predictable, high-precision direct fabrication of complex three-dimensional morphologies. However, the trade-off between processing efficiency and surface quality becomes especially pronounced in high-aspect-ratio structures. Managing scan paths and thermal accumulation in these structures is highly complex, necessitating the development of model-based adaptive scanning strategies. These approaches should include the integration of computational optical field or parallel multi-focus processing technologies, enhancing efficiency significantly without sacrificing quality.

Similarly to ablation processes, the ultimate outcomes of femtosecond laser direct writing in micro/nano fabrication are fundamentally determined by the synergistic effects of laser parameters. Fang et al. [67] conducted a systematic analysis of how parameters such as laser power and scanning speed influence the morphology of micro blind holes, revealing nonlinear relationships between these parameters and hole depth/diameter. In the pursuit of extreme precision, Gao et al. [68] employed a 343 nm femtosecond laser to fabricate linear nanostructures with a line width of approximately 61 nm on sapphire crystal surfaces, achieving processing precision beyond the optical diffraction limit. Qi et al. [69] and Pallarés-Aldeiturriaga et al. [65] also investigated the regulatory patterns of parameters on processing outcomes from the perspectives of trench quality and deep-etching schemes, respectively. The relationship between various parameters and scan counts in sapphire is shown in Figure 3E. Fang et al. [67] conducted systematic research on blind hole processing, providing a detailed process database and guiding principles for fabricating common structures such as micro-holes and micro-cavities. The phenomena they revealed, such as the saturation effect, are crucial for predicting processing limits. Gao et al. [68] achieved the pinnacle of precision in femtosecond laser processing by overcoming the diffraction limit through shorter wavelengths and precise parameter control, marking a milestone for next-generation nanophotonic device fabrication. Collectively, these studies demonstrate that femtosecond laser direct microfabrication is a highly nonlinear process, where minor variations in parameters can lead to significant differences in outcomes. The most valuable current achievement is the systematic mapping of the complex relationship between key process parameters and the resulting microstructure morphology. However, the fundamental challenge remains: how to precisely control electron dynamics and phase transitions within the processing zone to prevent the shift from “cold processing” to thermal damage. Potential solutions may involve developing novel laser sources, such as dual-wavelength lasers or spatiotemporally shaped pulses, to more effectively regulate energy deposition. This approach, combined with real-time imaging and spectral diagnostics, enables closed-loop control of the fabrication process.

Femtosecond laser direct writing in micro/nano fabrication demonstrates irreplaceable application value in high-end manufacturing. Elgohary et al. [70] employed femtosecond pulse laser technology to directly fabricate fluidic channels and achieve bonding on sapphire substrates, ultimately producing sealed microfluidic devices. Yuan et al. [71] leveraged femtosecond laser micromachining of nanostructures directly on fiber endfaces to fabricate reflection-based surface-enhanced Raman scattering fiber probes, demonstrating its potential in biochemical detection. The work by Elgohary et al. [70] fully demonstrates the capability of femtosecond lasers as a “one-stop” solution, where microchannel processing through to device bonding and encapsulation are all accomplished by the femtosecond laser. significantly streamlining the process flow while enhancing device integrity and reliability. This offers an ideal manufacturing approach for microfluidic systems operating in extreme environments. Yuan et al. [71] further highlighted the advantages of femtosecond lasers in functional structure fabrication, enabling the creation of intricate geometries and surfaces with enhanced optical properties. By ingeniously leveraging the inherently low background noise characteristics of sapphire fibers, researchers have successfully fabricated high-performance sensing probes. Currently, femtosecond laser direct microfabrication has evolved from a technique for creating simple into a platform for generating complex integrates optical, fluidic, mechanical, and other functions onto a single sapphire substrate. However, broader application remains primarily hindered by cost and efficiency challenges. Overcoming these obstacles will depend on the widespread adoption of high-power, high-repetition-rate femtosecond lasers and the advancement of manufacturing strategies such as parallel processing and intelligent path planning.

## 3. Surface and Internal Modification

The modification effects of femtosecond lasers on sapphire primarily result from unique physical processes triggered by the interaction of their ultrashort pulses with the material. At the surface level, the extremely high peak power, combined with nonlinear absorption mechanisms, enables precise removal of sapphire within timeframes much shorter than its thermal diffusion timescale. This allows the creation of structures at the micrometer or even nanometer scale with virtually no heat-affected zone, such as altering the damage threshold and the hydrophilic or hydrophobic properties of the sapphire surface. Internally, when focused within the sapphire crystal, the femtosecond laser enables localized modifications within the transparent medium by controlling pulse energy and focal depth. These modifications induce changes in photon absorption and reflection rates, opening possibilities for sapphire applications in integrated optical devices and microfluidic chip fabrication.

### 3.1. Surface Damage Threshold Regulation

**Figure 4:**
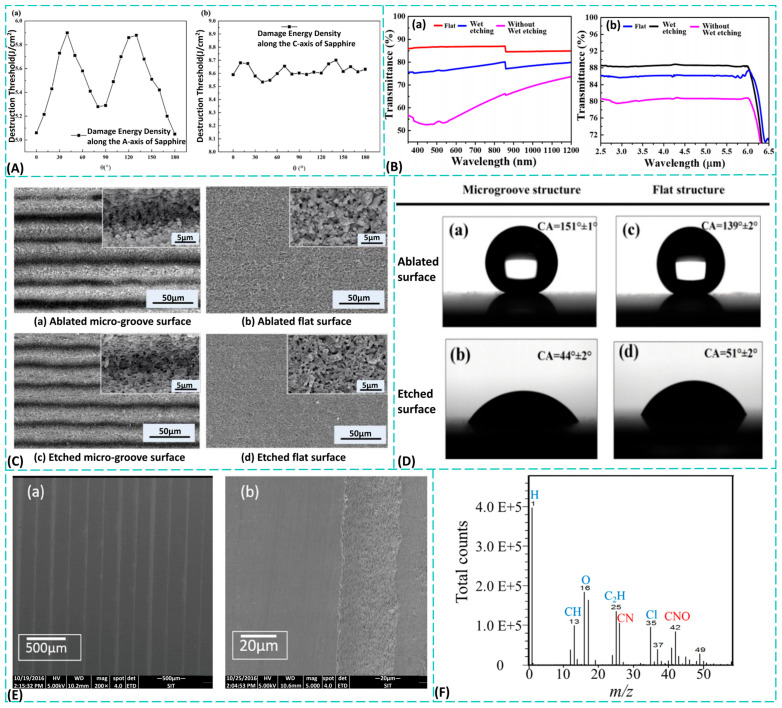
(**A**) Variation in sapphire surface damage threshold with θ when femtosecond laser incident along the (**a**) a-axis and (**b**) c-axis of sapphire [72]; (**B**) Transmission of micrometer-scale substructured sapphire [81]: (**a**) Transmission in the 350–1200 nm band, (**b**) transmission in the 2.5 µm–6.5 µm band; (**C**) Morphological features of prepared surfaces [86]: (**a**) laser-ablated microgroove surface, (**b**) laser-ablated flat surface, (**c**) etched microgroove surface, (**d**) etched flat surface; (**D**) presents measurement images of water contact angle (CA) under different surface treatment processes [86]: (**a**,**b**) show water droplet morphology on ablated microgroove surfaces and etched microgroove surfaces in air, respectively; (**c**,**d**) correspond to water droplet morphology on ablated flat surfaces and etched flat surfaces in air; (**E**) Scanning electron microscope (SEM) images of the microstructure of the striped U-shaped groove surface [88]: (**a**) at 200× magnification, (**b**) at 5000× magnification; (**F**) TOF-SIMS spectrum of the sapphire substrate irradiated at 0.8 W [90]. The detection ion was a negative ion.

When femtosecond lasers interact with sapphire surfaces, the damage threshold is not constant but is influenced by a combination of factors, including laser parameters and the material’s inherent properties. As the cumulative number of laser pulses increases, this threshold significantly decreases. This occurs because prior pulses induce micro- to nanoscale defects on the material surface or create a modified absorption layer, thereby reducing the energy required for subsequent pulses to cause damage. For instance, Niu et al. [72,73] employed damage area extrapolation and pump-probe shadow imaging to systematically investigate the spatiotemporal evolution of femtosecond laser-induced plasmas, revealing the influence of polarization direction on the damage threshold, as illustrated in Figure 4A. Additionally, Wen et al. [74,75] investigated femtosecond laser processing techniques on gold-coated sapphire, discovering that the gold film reduces ablation thresholds while improving ablation morphology. Yin et al. [76] reduced the threshold for LIPSS fabrication by depositing a gold nanolayer on sapphire surfaces, achieving more uniform periodic structures. The work of Niu et al. [72,73] provided critical insights into laser-material interactions in anisotropic crystals through plasma dynamics and polarization effects, where the periodic relationship between polarization angle and damage threshold offers theoretical guidance for anisotropic processing. Similarly, Wen et al. [74,75] and Yin et al. [76] demonstrated the potential of surface modification to enhance processing efficiency and quality, with the latter pioneering an approach for efficient surface structuring. Collectively, their findings highlight the combined influence of surface states and laser parameters on the damage threshold, underscoring the role of interfacial carrier enhancement in lowering this threshold.

Extending this discussion, the precise calibration and control of the damage threshold on sapphire surfaces under femtosecond laser irradiation establish a critical energy window and serve as a fundamental benchmark for achieving high-quality, reliable ultra-precision machining. However, a significant gap remains in existing studies: the lack of a quantitative description of the interfacial energy transfer mechanism, which makes precise prediction of damage thresholds challenging. Integrating first-principles calculations with experimental validation could establish a dynamic model of interfacial electron–phonon coupling, thereby guiding the design and optimization of surface modification layers. The practical application of this calibrated threshold is exemplified by work such as that of Vilar et al. [77], who employed femtosecond laser surface treatment to regulate the morphology and structure of sapphire. Using a 560-fs, 1030-nm wavelength laser on (0,1,2) plane sapphire wafers, they found that when the laser intensity was maintained slightly above the ablation threshold, self-assembled periodic structures with an average spatial periodicity of approximately 300 nm formed. This work by Vilar et al. [77] underscores both the potential and the precision control challenges of femtosecond laser micromachining. Future breakthroughs will therefore depend not only on advancements in laser technology but also on integrating deeper physical insights, intelligent control algorithms, and the convergence of multiple technologies to extend this powerful tool from the laboratory to broader industrial applications.

### 3.2. Regulation of Photon Absorption, Reflection, and Transmission Rates

Following an in-depth investigation of surface damage thresholds, we focused on the regulatory effects of femtosecond lasers on sapphire’s optical properties. Femtosecond laser irradiation induces changes in sapphire’s optical characteristics. This dynamic modulation process encompasses everything from initial transient interactions to the eventual formation of permanent microstructures. At the moment of laser exposure, the extremely high peak power leverages nonlinear multiphoton absorption mechanisms. This causes electrons in sapphire, originally transparent to specific laser wavelengths, to become excited within an extremely short time, resulting in a sharp surge in absorption rates and initiating the modification process. By precisely controlling parameters, femtosecond lasers can induce periodic rippled structures far smaller than the wavelength or fabricate micro/nano-cone arrays on the sapphire surface. These structures effectively capture photons, significantly reducing reflectivity in specific bands through multiple scattering and internal reflection. This achieves an anti-reflective effect similar to “black silicon” and even exhibits structural color phenomena. Further investigation is needed to deepen the understanding of the microscopic mechanisms involved. Wang et al. [78] constructed a finite element model for studying the non-thermal processes of sapphire femtosecond laser ablation. They observed a marked increase in surface absorption coefficient and reflectivity within picosecond timescales. Correspondingly, Cai et al. [79] simulated the transient nonlinear multiphoton absorption process in sapphire under femtosecond laser irradiation of varying wavelengths by solving the Fock-Planck partial differential equation and the TTM gradient coupling equation. They elucidated the wavelength-dependent patterns influencing ablation morphology. The simulation work conducted by both teams holds significant theoretical value, advancing femtosecond laser processing from macroscopic experimental observations to microscopic simulations of electronic dynamics. They clearly demonstrate the extremely shallow energy deposition characteristics and the decisive role of multiphoton absorption orders in determining the final morphology. This establishes a robust physical foundation for predicting processing outcomes and optimizing laser parameters. However, existing models face a bottleneck: their accuracy heavily depends on material properties under extreme conditions, which are often difficult to measure precisely. To address this, we can integrate experimental techniques such as ultrafast pump-probe spectroscopy to directly measure key parameters, including electron temperature and electron–phonon coupling coefficients, and then employ machine learning methods to calibrate and optimize the models.

Meanwhile, the impact on transmittance exhibits dual characteristics. On one hand, defects and scattering centers on the surface and within the material cause absorption and scattering losses, leading to a decrease in overall transmittance, particularly broadband transmittance. On the other hand, structures such as waveguides or Bragg gratings fabricated within the material via laser processing enable precise guidance and control of light paths at specific wavelengths, thereby optimizing functional transmittance for targeted applications. In functional optical device fabrication, Yue et al. [80] employed femtosecond laser direct writing to create maskless antireflective subwavelength structures, achieving dual objectives of infrared enhancement and visible light reduction on sapphire windows. Similarly focused on enhancing optical performance, Wang et al. [81] employed defocused femtosecond laser direct writing combined with high-fluoride wet etching to fabricate submicron structures on sapphire surfaces exhibiting high transmittance across a broad spectral range. They observed transmission variations as depicted in Figure 4B. Yue et al. [80] demonstrated the formidable capability of femtosecond laser processing in creating complex optical functions. Moving beyond mere “anti-reflection” or “anti-reflection enhancement,” they proactively and differentially modulated optical responses across different bands tailored to specific applications (infrared detection), embodying a “function-oriented” advanced manufacturing philosophy. Research by Wang et al. [81] further demonstrated the advantages of hybrid processing techniques. Combining defocused laser processing with wet etching achieved high transmittance and specific structures that would be difficult to attain with single-laser processing alone. These techniques enable the “tailoring” of sapphire’s optical properties (absorption, reflection, transmission) by designing surface micro/nano-structures. However, the primary challenge currently lies in the need for systematic evaluation of the mechanical strength, environmental stability, and long-term durability of these functional structures. To address this, we can deposit an ultra-thin, robust protective layer [82] (such as diamond-like carbon) on the surface of the structures or perform low-temperature annealing after processing to eliminate internal stresses and enhance structural stability.

### 3.3. Surface Hydrophilicity and Hydrophobicity Regulation

Femtosecond laser modification enables active regulation of sapphire surface wettability through precise control of surface microstructure and chemical composition. The core mechanism relies on nonlinear absorption effects to induce complex hierarchical micro-/nanostructures—such as periodic corrugations, nanoparticle arrays, or micro-cone arrays—which significantly increase surface roughness and provide the physical basis for tuning wettability. For example, Wang et al. [81] used defocused femtosecond laser direct writing combined with high-fluoride wet etching to create submicron structures exhibiting outstanding hydrophilicity. To enhance liquid transport, Yin et al. [83] developed a method for fabricating linear superwetting structures on sapphire using femtosecond lasers, enabling rapid water uptake and anti-gravity diffusion even on vertical surfaces. This advance shifts femtosecond laser processing from “static” surface property control to the manipulation of liquid “dynamic” behavior. The strong capillary forces and anti-gravity climbing capability demonstrated by such structures offer a key component for pump-free, self-driven microfluidic systems, with disruptive potential in microreactors, lab-on-a-chip devices, and electronic cooling. Initially, laser processing generates numerous dangling bonds on the fresh surface, which readily react with ambient water and hydrocarbons, often leading to enhanced hydrophilicity or even superhydrophilicity. This state can later be converted to a stable superhydrophobic state through chemical modification or natural aging. When micro/nano composite structures are coated with low-surface-energy substances (e.g., airborne adsorbates or applied fluorosilanes), a composite contact state analogous to the lotus effect is formed, trapping air within the roughness and yielding high contact angles. Achieving long-term stability remains a key challenge. Yan et al. [84] addressed this for superhydrophilic surfaces by combining femtosecond laser processing with a TiO_2_ coating that chemically coordinates with the substrate, suppressing adsorption of hydrophobic groups and maintaining superhydrophilicity for up to 180 days. Meanwhile, Xie et al. [62] and Chu et al. [85] demonstrated stable hydrophobicity and underwater superoleophobicity, respectively. The latter expands the application scope to marine anti-fouling and oil-water separation. These studies underscore the synergy between femtosecond laser structuring and subsequent chemical modification in creating stable functional surfaces. A current frontier is multifunctional integration—e.g., realizing stable superhydrophilicity, superhydrophobicity, and underwater superoleophobicity on distinct regions of a single substrate. This may be achieved by leveraging the spatial selectivity of femtosecond lasers for zoned, stepwise processing and modification.

Applications initially focused on self-cleaning, where superhydrophobic micro/nano structures on sapphire allow water droplets to roll off and carry away contaminants, benefiting optical windows and sensors in harsh environments. In microfluidics, femtosecond lasers can define hydrophilic and hydrophobic regions within channels on sapphire, enabling valve-free guidance, directional transport, and precise stream splitting without chemical coatings, thereby simplifying chip fabrication and improving reliability. Designed wettability patterns also facilitate cell biology research, e.g., confining cell growth to hydrophilic microarrays while preventing adhesion in surrounding superhydrophobic zones for high-precision cell positioning and culture. To achieve such precise control, Liu et al. [86] combined femtosecond laser irradiation with chemical etching to texture sapphire surfaces. They produced four microstructures (Figure 4C), finding that the ablated surface was hydrophobic, with microgrooves reaching superhydrophobicity (contact angle 151° ± 1°), while the etched surface showed hydrophilic behavior (Figure 4D). This composite approach overcomes the limitations of single-step laser processing, enabling cross-domain wettability regulation and offering new routes for functionalizing hard, brittle materials. Nevertheless, challenges remain in balancing processing efficiency with micro-/nanostructure uniformity, and in fully elucidating the quantitative links between surface chemistry and wettability. Future work may explore dynamic control strategies—such as multi-level composite structures or stimulus-responsive materials—to evolve surfaces from static wettability toward dynamic, intelligent responsiveness.

### 3.4. Regulation of Lattice Structure and Mechanical-Chemical Properties

Beyond the widespread applications of femtosecond lasers in modifying sapphire surfaces and internal structures, these lasers also significantly affect the crystal structure of sapphire. This process is not a simple thermal melting but involves a complex sequence of dynamics, ranging from electron excitation to the ultimate modification of the crystal lattice. When a femtosecond laser is focused onto sapphire, its extremely high peak power, through nonlinear multiphoton absorption processes, instantaneously deposits energy into the electron subsystem on a timescale much shorter than the electron–phonon coupling time. This causes the electron temperature in the irradiated region to rise sharply, while the crystal lattice remains relatively cold. This extreme non-equilibrium state triggers intense Coulombic repulsion, sufficient to disrupt the periodic arrangement of aluminum-oxygen bonds. As a result, high-density point defects, color centers, and dislocations are generated within the lattice. Zhao et al. [87] employed ultrafast pump-probe techniques to demonstrate that intense femtosecond laser irradiation induces defects, distortions, and even recrystallization in sapphire crystal structures. Xu et al. [88] observed that gallium nitride epitaxial layers grown on sapphire substrates patterned with femtosecond laser ablation stripes exhibited significantly enhanced crystallinity, as evidenced by surface topography (Figure 4E). Femtosecond lasers offer a powerful method for achieving controlled, localized modifications of sapphire crystal structures, from point defects and amorphization to periodic nanostructures, establishing the physical foundation for applications in integrated optics, three-dimensional data storage, and sensors designed for extreme environments.

Regarding the mechanical properties of sapphire, femtosecond lasers can locally enhance the strength and toughness of sapphire surfaces by inducing surface nanocrystallization or generating residual compressive stress layers. Additionally, their precise modification capabilities can be used to pre-introduce controllable microcracks or stress concentration points in specific regions, thereby guiding subsequent fracture paths and enabling high-quality, low-damage cutting of sapphire wafers. Cao et al. [89] utilized femtosecond lasers to fabricate micro/nano structures on sapphire surfaces. By forming structural interlocking with metals, they significantly enhanced the bonding strength of copper/sapphire joints. This interface strengthening method, employing femtosecond lasers to create surface micro/nano structures for structural interlocking, offers a novel approach for bonding sapphire with metals, thereby advancing device integration processes.

Femtosecond laser processing creates active defects and a large specific surface area, which significantly enhance the chemical reactivity of the sapphire surface. This increased reactivity makes the surface more susceptible to selective etching by acidic or alkaline solutions. Miyagawa et al. [90] observed the energy spectrum of sapphire substrates irradiated at a power of 0.8 W (Figure 4F) and detected a high concentration of nitrogen ions in the irradiated area at this power. They found that irradiating sapphire with a femtosecond laser in a nitrogen atmosphere can form nitrogen-related chemical bonds on its surface, thereby altering its chemical properties. Liu et al. [91] developed a femtosecond laser etching method for sapphire, achieving high etch rates at room temperature while reducing process requirements. They observed that femtosecond laser modification substantially enhances etch rates and investigated the influence of processing parameters. This technique holds potential applications in integrated optics and microfluidic devices. Jiang et al. [92] compared changes in YAG and sapphire after femtosecond laser irradiation. Sapphire darkened due to microcracking caused by self-focusing effects, while YAG darkened through color center formation. Analysis of micrographs and absorption spectra confirmed that TGT-grown sapphire outperformed CZ-grown sapphire, and YAG exhibited superior irradiation resistance compared to sapphire. The most significant achievement of these studies lies in revealing the distinct interaction mechanisms between femtosecond lasers and different transparent brittle materials, specifically, color center formation in YAG versus microcracking in sapphire. This insight provides crucial guidance for optimizing laser processing parameters and selecting appropriate material systems (e.g., preferring TGT-grown sapphire) to enhance device performance and processing yield. By integrating laser sources with shorter pulses and higher repetition rates, developing multi-step processing techniques tailored to material properties (e.g., modification followed by etching), and employing simulations to predict laser-material interactions in advance, more precise control over processing outcomes can be achieved.

## 4. Optical Waveguide Writing

**Figure 5:**
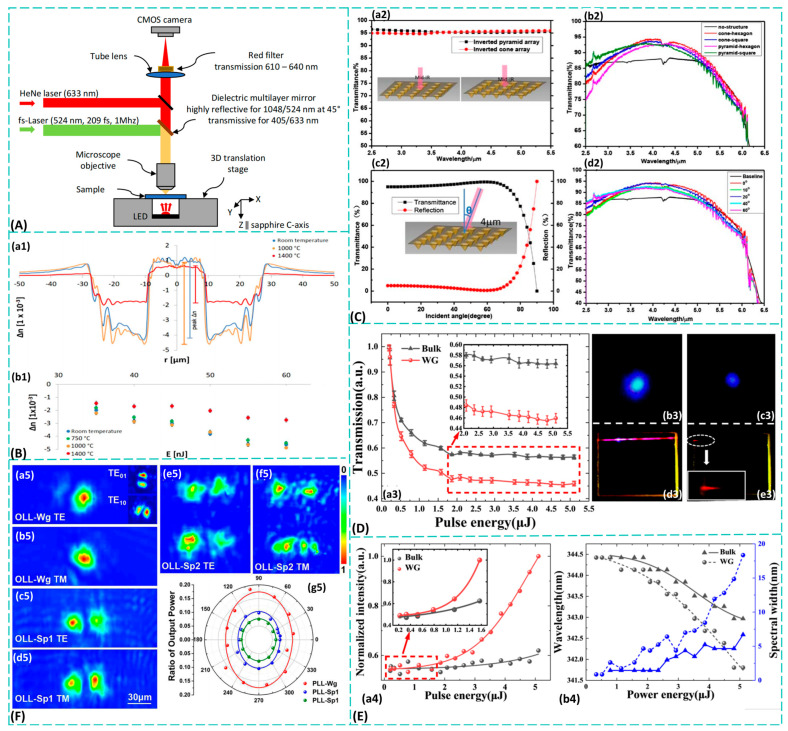
(**A**) Optical setup of the laser lithography platform [94]. A fs-laser is widened and collimated before it enters the setup. Here it is superimposed with a HeNe laser, which is visible in the imaging setup and used for z-levelling. The sapphire crystal C-axis is parallel to the z-axis in this setup. (**B**) shows (**a1**) the radial refractive index distribution curve measured after continuous annealing cycles, and (**b1**) the trend of DCW peak refractive index versus pulse energy at different annealing temperatures [95]; (**C**) (**a2**) Theoretical simulation of the designed SWSs transmittance, (**b2**) measured transmittance of fabricated SWSs, (**c2**) Theoretical simulation of the relationship between incident angle and transmittance (incident wavelength = 4 μm), (**d2**) Measured transmittance versus incident angle curve [96]; (**D**) (**a3**) Dependence of transmittance versus input energies, measured at λ = 1036 nm for both a 5 mm WG and sapphire bulk. (**b3**,**c3**) are the spot obtained on the white paper for the outgoing light from the waveguide and bulk, respectively. (**d3**,**e3**) Side images of the waveguide and bulk, respectively. Note: WG stands for the waveguide [98]; (**E**) (**a4**) Spectral integrated intensity versus input pulse energy measured in the 300–400 nm range when the integration time of the spectrometer is 10 ms. The inset is the variation of the spectral integrated intensity measured in the same wavelength range with the input pulse energy of 0.2 µJ–1.57 µJ and a spectrometer integration time of 500 ms. The left and right axes in (**b4**) show the variation of the TH peak position and the corresponding spectral width with the input pulse energy (0.20 µJ–5.10 µJ), respectively [98]; (**F**) (**a5**–**f5**) The measured TE and TM mode distributions of OLL-structures at 1064 nm. The insets of (**a5**) show TE01 and TE10 modes of straight forward waveguide OLL-Wg. (**g5**) Polarization dependency of OLL-structures. The red, blue and green balls represent the experimental results for OLL-Wg, OLL-Sp1 and OLL-Sp2, respectively. The solid lines are elliptical fit of experiment data [104].

The core principle of optical waveguide writing relies on the extremely high peak power of femtosecond lasers, which is particularly advantageous for processing robust materials like sapphire. Sapphire is a critical substrate for integrated photonics in extreme environments due to its exceptional hardness, broad optical transparency, and outstanding chemical and thermal stability—offering significant advantages over conventional materials like silicon or glass for applications involving high temperatures, pressure, or corrosive conditions. Through nonlinear effects such as multiphoton absorption, this power induces permanent modifications within an extremely small focal spot inside the crystal, while the surrounding regions remain largely unaffected. During processing, a precisely focused femtosecond laser beam scans along a predetermined three-dimensional path within the sapphire. The interaction between the laser and the material causes a small but critical increase in the refractive index of that region, thereby forming a channel capable of confining and guiding light transmission—an optical waveguide. Gross et al. [93] first achieved direct laser writing of waveguides in bulk Ti^3+^:sapphire using an ultrafast oscillator, significantly reducing fabrication time. The stress field generated by laser-induced damage zones within the crystal increases the refractive index, creating waveguides with pronounced birefringence. Each pair of damage lines simultaneously guides two polarized light waves, one polarized parallel and the other orthogonal to the crystal surface, whose propagation characteristics can be analyzed through near-field distribution and insertion loss measurements. Winkler and others [94] have used the optical setup of a laser lithography platform to write optical waveguides, as shown in Figure 5A. The writing device is placed on a granite base to minimize the impact of external vibrations. The optical components (including the focusing optical system and optomechanical elements) are mounted on a movable breadboard, and the focus of the writing beam is adjusted via a z-axis platform.

### 4.1. Fabrication of Waveguides

A photonic waveguide is a physical structure designed to confine and guide light waves along a specific path, operating primarily on the principle of total internal reflection. It typically consists of a core medium with a higher refractive index, surrounded by a cladding medium with a lower refractive index. This configuration effectively controls the propagation of light, significantly reducing diffusion losses during transmission, and enables various functions such as bending light paths, beam splitting, and coupling. Optical waveguides come in diverse forms, ranging from planar waveguides integrated onto chip surfaces to three-dimensional strip waveguides embedded within materials, as well as flexible optical fibers. They serve as fundamental components in modern integrated photonics, optical communications, and optoelectronic chips. For example, the fiber-optic networks that form the backbone of our daily internet communications are essentially cylindrical optical waveguides.

Femtosecond laser-fabricated waveguides find extensive applications in sapphire materials, with infrared waveguide fabrication being a common example. To achieve low-loss waveguides, Bérubé et al. [95] utilized femtosecond pulses at 515 nm to etch mid-infrared waveguides in sapphire, marking the first successful fabrication of low-loss mid-infrared waveguides in sapphire. These waveguides remained stable at high temperatures up to 1000 °C, with the resulting refractive index distribution shown in Figure 5B. Separately, Li et al. [96] pioneered the use of laser ablation combined with wet etching to fabricate mid-infrared surface-wave superstructures (SWSs) on sapphire surfaces. These structures achieved transmission spectra exceeding 90% across the 3–5 μm wavelength range, with the transmission trend illustrated in Figure 5C. Their work extended sapphire photonics research into the critical mid-infrared band. The revolutionary stability of the waveguides fabricated by Bérubé et al. [95] reported that photonic sensing and signal transmission in extreme high-temperature environments such as aircraft engines and nuclear reactors could be enabled at 1000 °C Li et al. [96] demonstrated that metasurface waveguides represented an alternative technical approach, transforming the “intra-body” light confinement of traditional waveguides into “surface” light modulation. This approach offers greater design flexibility and better compatibility with planar fabrication processes. These achievements demonstrate that femtosecond lasers can fabricate low-loss optical waveguides in sapphire suitable for harsh environments (high temperatures, strong corrosion). However, the primary technical bottleneck lies in further reducing transmission losses in waveguides, particularly in curved waveguides [97], to achieve higher-density photonic integration. Potential approaches include optimizing the repetition frequency and scanning speed of laser writing to minimize defects, and designing novel waveguide structures (such as recessed cladding waveguides) to enhance light confinement capabilities.

A concave cladding waveguide is a specialized integrated optical waveguide structure characterized by a cladding region engineered to have a lower refractive index than the surrounding substrate material. The cross-section of this structure exhibits a distinctive “sandwich” morphology: a circular or elliptical core channel, formed by laser-induced refractive index enhancement, lies at the center, flanked above and below by two “depressed” cladding regions. These regions, created using different processing parameters (such as higher pulse energy or varying scan speeds), have a refractive index lower than that of the original substrate. Winkler et al. [94] first measured the recessed cladding structure of visible-light waveguides in sapphire using femtosecond laser etching. Building upon this, Li et al. [98,99] successfully fabricated recessed cladding waveguides on sapphire crystals using a femtosecond fiber laser, discovering significantly enhanced nonlinearity compared to bulk materials. The recessed cladding waveguide structure exemplifies the essence of femtosecond laser writing technology. By meticulously designing laser scanning strategies, it creates a lower-refractive-index cladding around the waveguide core, thereby achieving more effective confinement of the optical mode field. As shown in Figure 5D, the nonlinear losses of the waveguide increase markedly with input pulse energy, significantly exceeding those of the bulk material, clearly demonstrating enhanced nonlinear effects within the waveguide structure. The nonlinear enhancement observed by Li et al. [98,99] is particularly significant, implying that nonlinear optical devices such as miniature lasers and frequency converters based on such waveguides can outperform bulk materials, paving the way for the development of integrated nonlinear photonic chips. Moreover, the enhanced nonlinearity is further corroborated by the pronounced blueshift of the third harmonic, illustrated in Figure 5E, where the waveguide exhibits a greater spectral shift compared to the bulk under the same input energy. While researchers have developed waveguide structures with high refractive index contrast and demonstrated the beneficial effects of waveguide confinement on enhancing nonlinear optical phenomena, challenges persist in precisely controlling the refractive index variation and spatial distribution within the cladding region to achieve ideal single-mode transmission and mode field matching. To address this, we propose integrating three-dimensional refractive index tomography [100] for non-destructive characterization of fabricated waveguides, enabling reverse optimization of laser processing parameters.

The primary advantage of the recessed cladding waveguide lies in its ability to achieve a higher refractive index contrast, enabling stronger and tighter confinement of the optical field. This not only significantly reduces bending loss, allowing for more compact curved optical paths, but also improves the waveguide’s resistance to interference and enhances its mode field characteristics. As a result, better mode field matching with standard optical fibers is achieved, reducing coupling loss. This makes it highly valuable for photonic chips requiring high-density integration and low-loss transmission. Ren et al. [101] successfully fabricated titanium-sapphire cladding waveguides using femtosecond laser inscription (FLI)—a precise, maskless technique that uses ultrafast laser pulses to directly write waveguide structures inside transparent materials. They systematically studied the waveguides’ guiding and fluorescence properties, demonstrating that this structure supports orthogonal polarization states over a 4 nm wavelength range while maintaining stable Ti^3+^ fluorescence in the waveguide region. Through broadband waveguide fluorescence emission experiments, they achieved a remarkable maximum slope efficiency of 3.5 × 10^4^, representing a tenfold improvement over the fluorescence performance of femtosecond laser-etched waveguides [102]. The excellent performance of this low-cladding structure makes it a promising integrated light source for biomedical applications such as optical coherence tomography. Moreover, such cladding waveguides show potential for generating tunable waveguide lasers without dependence on pump polarization. The breakthrough of this work lies in achieving an order-of-magnitude enhancement in fluorescence efficiency and polarization-independent lasing capability via FLI, providing key technical support for integrated broadband light sources.

### 4.2. Fabrication of Waveguide Splitters

Optical waveguide splitters are essential components in integrated photonic circuits. They distribute input optical signals into two or more distinct output waveguide channels according to specific proportional and phase relationships, enabling precise optical power splitting and routing. These devices are typically realized through the precise design of waveguide structures, with the most common configuration being the Y-branch, where a single input waveguide smoothly divides into two output waveguides. By carefully controlling the dimensions, shape, and taper profile at the branching point during fabrication, scattering losses can be minimized while achieving the desired splitting ratios. Ren et al. [103,104] successfully fabricated an optical waveguide splitter in titanium sapphire crystal, achieving a 1:2 equal split with low polarization dependence of the incident light and well-preserved luminescence characteristics within the waveguide. As shown in Figure 5F, the splitter exhibits clear dual-channel output profiles under both TE and TM polarizations, with near-Gaussian mode distributions, confirming its excellent splitting performance and polarization-independent behavior. Splitters are indispensable fundamental units in integrated photonic circuits. This research demonstrates the capability of femtosecond lasers to simultaneously integrate optical paths and functional elements within active crystal materials, marking a significant step toward realizing complex “labs-on-a-chip.” Future work may extend similar functionality to more complex materials (e.g., undoped sapphire [105]) and structures (e.g., multi-port beam splitters, Mach-Zehnder interferometers [106]).

## 5. Grating Fabrication

A grating is an optical element characterized by a precisely periodic structure. Its primary function is to modulate incident light through spatially periodic features, mainly utilizing diffraction effects to spatially separate different wavelengths within composite light. This periodic structure can appear as engraved surface lines or as periodic modulation of the refractive index within the material. Femtosecond lasers, exploiting nonlinear effects such as multiphoton absorption induced by their extremely high peak power, can create permanent periodic refractive index modulations or surface topographies within or on the surface of sapphire, thereby forming gratings. To enhance grating fabrication quality, Zheng et al. [107] employed femtosecond laser processing combined with wet etching. This approach effectively addresses issues such as poor surface quality and debris accumulation following direct laser writing, reducing grating roughness from 78 nm to just 7 nm. The researchers successfully fabricated microstructures with an 800 nm period and a 4:1 aspect ratio, significantly enhancing diffraction efficiency. The most valuable contribution of this study lies in combining laser processing with chemical etching, thereby overcoming the trade-off between surface quality and aspect ratio inherent in standalone femtosecond laser processing. This method not only substantially improves the optical performance (diffraction efficiency) of the grating but also establishes a universal hybrid processing strategy. Currently, pure femtosecond laser ablation faces precision limitations when fabricating high aspect ratio structures due to challenges such as melt redeposition and microcrack formation. Future research can further explore the optimal combinations of etching agents and laser parameters to achieve more precise three-dimensional control over structural morphology and extend this technology to more complex photonic crystal structures.

Building upon this foundation, Kim et al. [108] and Halstuch et al. [109] investigated the formation mechanisms of nanogrids. Kim et al. [108] fabricated polarization-dependent nanogrids with a period of approximately 250 nm on sapphire surfaces by controlling laser power, revealing that their formation is associated with surface plasmon standing waves and dielectric constant variations. Halstuch et al. [109] achieved the fabrication of similar structures on gallium nitride films. These studies provide a comprehensive understanding of the physical mechanisms underlying nanostructure formation through self-organization during ultrafast laser-matter interactions. This advancement paves the way for nanomanufacturing, eliminating the need for complex and costly peripheral optical systems. However, ensuring the reliability and consistency of such self-assembly processes remains challenging, as minor parameter fluctuations can cause significant structural variations. By integrating real-time imaging with feedback control systems to monitor plasma plume or scattered light signals during processing, it becomes possible to dynamically adjust laser parameters. This approach enables active control over the nanograting period and orientation, facilitating their transition from laboratory phenomena to practical applications.

Beyond traditional linear gratings, special-function gratings have attracted considerable interest. As a specially designed diffractive optical element, the Fresnel grating ingeniously merges the dispersive ability of conventional gratings with the focusing properties of a Fresnel zone plate. Its core structure does not consist of traditional straight, equally spaced lines, but rather a series of concentric annular rings. The spacing of these rings varies according to the principles of Fresnel zone plates, enabling the grating to perform both spectral dispersion and focusing functions. Li et al. [110] successfully fabricated a Fresnel zone plate (FZP) on a sapphire substrate. This integrated device combines spectral separation and focusing capabilities, demonstrating excellent ultraviolet imaging performance. The sapphire substrate ensures long-term stability under harsh environmental conditions. These advantages make sapphire-based FZPs promising ultraviolet micro-optical devices for practical applications. For example, arrays of FZPs on sapphire can be used in high-efficiency gallium nitride blue-ultraviolet LEDs [111]. Owing to these features, Fresnel gratings are highly valuable in applications that demand miniaturization and integration, playing a key role in fields such as miniature spectrometers, space remote sensing, and portable optical analysis devices.

Among the various types of gratings, the Bragg grating is the most widely used and significant. It is an optical device created by introducing periodic modulation of the refractive index within a medium, operating based on the principles of light interference and Bragg diffraction. When broadband light encounters this periodic structure, only wavelengths that satisfy specific phase-matching conditions are strongly reflected, while other wavelengths pass through. This enables selective filtering of light wavelengths. These unique optical properties make Bragg gratings essential in fields such as fiber optic communications, lasers, and sensors. Grobnic et al. [112] first fabricated an FBG within sapphire fiber, maintaining stable performance at 1500 °C. This milestone demonstrated, for the first time, that femtosecond lasers can fabricate fiber Bragg grating sensors within sapphire fibers capable of withstanding extreme temperatures, thereby directly extending the upper application temperature limit of fiber sensing beyond 1500 °C. This breakthrough provides unprecedented tools for in situ monitoring in fields such as aerospace and energy metallurgy.

Halstuch et al. [113] used a NIR femtosecond laser and phase mask to etch grating structures with a uniform periodicity of approximately 1.07 μm onto the surfaces of sapphire substrates, fused silica, and GaN films grown on sapphire substrates, without damaging the underlying sapphire substrates. They discovered that the ablation threshold for patterning gratings on GaN films was nearly one order of magnitude lower than that for refractive index gradient gratings in sapphire and fused silica. This method holds promise for future grating patterning in GaN waveguides for spectral filtering and mirror applications. Chen et al. [114] successfully fabricated large-diameter surface plasmonic fiber high-order Bragg gratings using femtosecond laser scanning exposure technology to explore the application potential of surface plasmonics in smart composites and embedded fiber sensors. By analyzing the multimode Bragg resonance spectra from the 4th to 8th orders, the researchers found a strong agreement between numerical simulations and experimental results at a coupling coefficient of 0.0020. However, when the coupling coefficient exceeded 0.0005, the resonance signals of each mode significantly overlapped, exhibiting distinct response characteristics for different higher-order resonances. Notably, the low-threshold grating etching capability of GaN films opens new avenues for integrating GaN optoelectronic devices. Additionally, the precise modulation of mode responses by coupling coefficients in higher-order Bragg gratings highlights the potential of femtosecond lasers for controlled processing of micro/nano photonic structures. The most significant breakthrough of their research is the achievement of non-destructive processing of submicron structures on wide-bandgap material surfaces. Furthermore, by quantitatively correlating simulations with experiments through coupling coefficients, they provide a solid experimental foundation for designing complex photonic devices. Nevertheless, the study identifies persistent challenges, such as narrow processing windows (e.g., balancing low thresholds in gallium nitride with substrate protection) and multimode coupling crosstalk. Future efforts may require developing methods that combine real-time plasma monitoring with adaptive optical field control. By dynamically adjusting laser parameters to achieve closed-loop control during processing, the consistency and functional integration of complex micro/nano photonic structures can be significantly enhanced.

**Figure 6:**
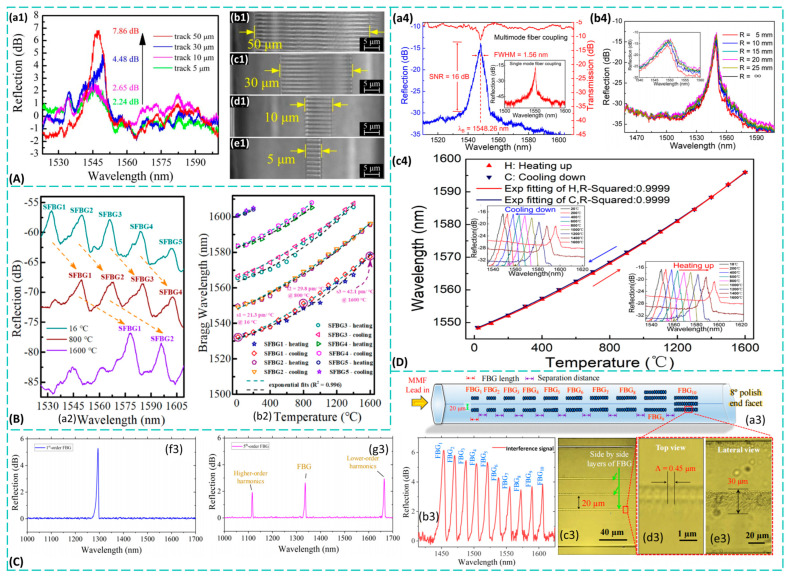
(**A**) Reflection spectra of four SFBGs inscribed with increasing track lengths L [116]. (**a1**) Reflection spectra of these SFBGs. (**b1**–**e1**) Microscope images of the top view of the SFBGs with different track lengths of 50 μm, 30 μm, 10 μm, and 5 μm, respectively. (**B**) (**a2**) Evolutions of reflection spectra of the WDM SFBG array at elevated temperatures of 16 °C, 800 °C, and 1600 °C. Hereafter, the spectra are plotted with an offset in the vertical axis for clarity. (**b2**) Bragg wavelengths of the serial SFBG array as functions of the temperature in the case of temperature cycling from 16 °C to 1600 °C [115]. (**C**) Proposed sapphire optical FBG-based distributed sensors [122]: (**a3**) schematic overview of the sensor configuration; (**b3**) reflection spectrum of the 10 FBGs; (**c3**) microscopy image of FBG10; (**d3**) enlarged image of FBG10 with a grating period of 450 nm (top view); (**e3**) lateral view of FBG10, highlighting the depth of the laserirradiated reflector; and the reflection spectra of the point-by-point (**f3**) first-order FBG and (**g3**) fifth-order FBG. (**D**) (**a4**) Reflectance and transmittance spectra of HSFBG obtained via multimode fiber coupling, with an inset showing the reflectance spectrum of HSFBG obtained via single-mode fiber coupling; (**b4**) Spectral response of HSFBG at different bending radii. The illustration is a partial enlarged view in the range of 20 nm; (**c4**) Temperature-dependent evolution of HSFBG’s Bragg wavelength during cooling and heating processes [124].

The performance of Bragg gratings is heavily influenced by the fabrication technology employed. Primary methods such as point-by-point etching, line-by-line scanning, and planar writing—collectively referred to as femtosecond laser direct writing—enable the creation of gratings in diverse materials and support the design of specialized structures with complex cut-off, chirped, or tilted profiles. Researchers including Xu [115,116], Alla [117], and Guo [118] have compared point-by-point and line-by-line techniques, focusing on optimizing spectral characteristics like reflectivity and bandwidth. Xu [116] and others, in their study on fabricating sapphire fiber Bragg gratings through line-by-line scanning, systematically investigated the effect of trace length on reflectivity. The results are shown in Figure 6A, which illustrates how longer traces enhance reflectivity by increasing the area of refractive index modulation. He et al. [119] developed a cylindrical telescope-assisted approach that reduced fabrication time to just 20 s, greatly improving efficiency. The most widely applied sensor resulting from these efforts operates stably at extreme temperatures up to 1600 °C, Figure 6B shows the Bragg wavelength shift of the SFBG array from room temperature to 1600 °C, demonstrating its stable and nonlinear thermal response [115], opening avenues for in-situ monitoring in fields such as aircraft engines and nuclear reactors. A core challenge in this area remains achieving repeatable, high-performance mass production of sapphire fiber gratings. While point-by-point and line-by-line scanning offer flexibility, their low throughput and demanding motion-control requirements drive the search for alternatives. Potential solutions include multi-beam parallel processing using diffractive optical elements or spatial light modulators to write multiple gratings simultaneously, or leveraging nonlinear processes such as femtosecond laser filamentation [120] to form gratings in a single exposure, thereby eliminating the need for precision scanning.

Since the fabrication of Bragg gratings primarily involves physical modifications rather than conventional photosensitive chemical reactions, the resulting gratings typically exhibit superior thermal and mechanical stability, making them well-suited for sensing applications in high-temperature and harsh environments. Mumtaz et al. [121,122] employed femtosecond laser-assisted line-by-line scanning and point-by-point etching techniques, respectively, to achieve cascading and co-located integration of multiple FBGs within sapphire fibers, as illustrated in Figure 6C, which demonstrates the reflective spectrum and microscopic images of the point-by-point fabricated FBG array, highlighting the high-quality fringe visibility achieved through this method. This approach effectively resolves cross-sensitivity issues between temperature and strain, overcoming the challenges of simultaneous multi-parameter measurement under harsh conditions. Current challenges in multi-grating integration include crosstalk and packaging. Future advancements will depend on AI-assisted signal processing algorithms to accurately extract parameter information from complex spectral responses. Combined with advanced laser 3D processing techniques, this approach will enable the integrated fabrication of sensing gratings, waveguides, and even microfluidic channels within a single sapphire fiber, creating true “lab-on-a-chip” sensors. He et al. [123] employed femtosecond laser direct writing techniques (including point-by-point, line-by-line, and planar writing) to flexibly fabricate SFBGs with varying Bragg wavelengths. The manufacturing process incorporated filamentation and cylindrical telescope technologies, effectively enhancing production efficiency and spectral characteristics. They discovered that the broadband reflection spectrum of SFBGs originates from multimode operation, significantly affecting sensing performance. For single-mode SFBGs fabricated using a helical structure, the bandwidth was reduced to 0.18 nm with a reflectance as high as 66.3%. Guo et al. [124] fabricated Gaussian fiber Bragg gratings (HSFBG) within 60-µm-diameter sapphire fibers using a 515-nm femtosecond laser direct-writing system. They tested performance parameters including reflectance and transmittance measurements, bending tests, and wavelength-temperature response, with the results shown in Figure 6D. These studies have made significant progress in the flexible fabrication and performance optimization of special-structure fiber gratings using femtosecond laser direct writing technology. Researchers effectively enhanced fabrication efficiency and spectral characteristics of SFBGs through filamentation and cylindrical telescope techniques. The achieved 0.18-nm narrow bandwidth and 66.3% high reflectivity demonstrate the potential for precise control of grating performance under single-mode conditions, which is highly valuable for high-precision sensing applications.

## 6. Welding and Joining

**Figure 7:**
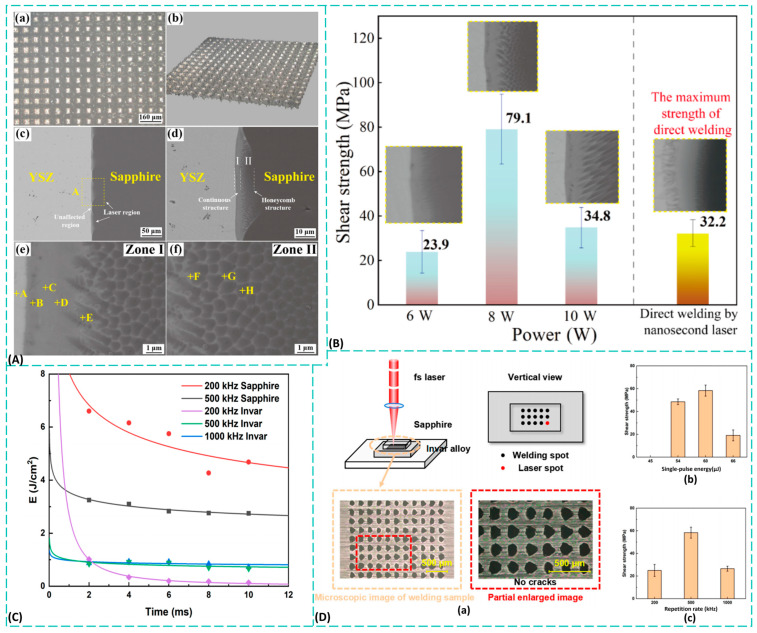
(**A**) Macroscopic and microstructure of YSZ/sapphire joint welded by femtosecond laser [125]: (**a**,**b**) optical morphology of YSZ/sapphire joint; (**c**–**f**) microstructures of YSZ/sapphire joint. (**B**) Shear strength of YSZ/sapphire joint under different laser powers [125]. (**C**) Multi-pulse ablation threshold fitting results for sapphire and Invar alloy at different repetition rates [129]; (**D**) Experimental results of ultrafast laser single-spot welding of the sapphire/Invar alloy [129]. (**a**) Schematic representation of the single-spot welding process; (**b**) measured shear strength at different pulse energies; (**c**) measured shear strength at different repetition rates.

Femtosecond laser welding of sapphire to metals enables the creation of high-strength, hermetic connections between sapphire and various metals such as aluminum, titanium, and stainless steel. This technique expands sapphire’s applications and imparts superior properties to the resulting composites. Thanks to the femtosecond laser’s exceptional spatial selectivity, the entire process achieves extremely high precision and flexibility, providing a reliable solution for the encapsulation and manufacturing of high-performance devices in fields such as microelectronics, aerospace, and biomedical engineering. A critical factor in the welding process is the joint strength at the welded interface, which determines whether the components will function properly. Currently, many researchers are exploring various methods to enhance joint strength. Zhang et al. [125] utilized a femtosecond laser with a titanium metal interlayer to achieve high-strength micro-welding between Yttria-stabilized Zirconia (YSZ) and sapphire. They also made the first discovery that the honeycomb structure formed at the interface is key to enhancing joint strength (Refer to the typical honeycomb structure at the interface in Figure 7A). In addition, under the 8 W laser power condition, the joint exhibits the best interfacial performance, with a maximum shear strength of about 79 MPa, as shown in Figure 7B. Similarly, Du et al. [126] systematically investigated the indirect brazing process between sapphire and zirconia using femtosecond laser texturing. They investigated enhancing the performance of dissimilar ceramic brazed joints between sapphire and zirconia (ZrO_2_) by creating periodic microstructures on the sapphire surface using a femtosecond laser. Periodic micro-cone arrays were fabricated on the sapphire surface to increase the interfacial reaction area and improve mechanical interlocking. A metal layer was pre-coated on the sapphire surface using Ag-Cu-Ti paste to promote flux wetting and interfacial bonding. The brazing connection between sapphire and ZrO_2_ was completed in a vacuum furnace using Ag-Cu-Zn-Sn brazing filler metal. Experimental comparisons revealed that laser texturing formed a TixOy reaction layer on the sapphire surface, which reduced the formation of brittle Ti-Cu intermetallic compounds. The shear strength of the laser-treated joint reached 158 MPa, representing approximately a 50% increase compared to the untreated joint (103 MPa). Ultimately, femtosecond laser surface texturing was found to significantly enhance the microstructure and mechanical properties of sapphire/ZrO_2_ brazed joints. Their research revealed a unique mechanism by which femtosecond laser welding surpasses conventional thermal welding. It relies not only on metallurgical reactions but also generates powerful “structural interlocking” effects by inducing specialized microstructures at the interfaces (e.g., honeycomb structures, micro-cone arrays). This synergistic interaction between mechanical anchoring and chemical bonding is fundamental to achieving high-strength connections. It also demonstrates that femtosecond lasers can actively “design” and “enhance” joint strength by controlling interfacial microstructures, rather than passively relying on inherent material properties. However, the primary challenge in the welding process lies in balancing the avoidance of sapphire cracking (due to its vastly different thermal expansion coefficient from metals) with ensuring sufficient interfacial reaction. We can employ ultra-short pulse, low-repetition-rate laser parameters to minimize thermal input and explore nanocomposite interlayer materials with transitional thermal expansion coefficients [127] to buffer thermal stresses.

Invar alloy is an alloy composed of iron (Fe) and nickel (Ni), primarily consisting of 36% nickel and 64% iron. Its notable characteristic is an extremely low thermal expansion coefficient, resulting in minimal dimensional change under temperature variations. This property enables its extensive application in precision instruments and high-tech products. Pan et al. [128] successfully achieved the first direct connection between sapphire and Invar alloy using femtosecond laser micro-welding technology. This research demonstrates that femtosecond laser micro-welding serves as a convenient, flexible, and viable method for direct bonding between sapphire and metals, offering high precision, strength, and efficiency. Building upon this initial Invar alloy welding achievement, Chen et al. [129] systematically investigated methods for achieving high-quality connections between sapphire and Invar alloy using femtosecond laser single-point welding under non-optical contact conditions. By establishing a two-temperature model for femtosecond laser-Invar alloy interaction and simulating electron and lattice temperature evolution, they determined ablation threshold curves for sapphire and Invar alloy at different repetition rates using the D2 method (results shown in Figure 7C). They then employed femtosecond laser single-point welding (non-scanning) to optimize welding parameters by controlling pulse energy and repetition rate, and the corresponding welding strength results are shown in Figure 7D. Similarly, for Invar alloy, Li et al. [130] primarily investigated effective micro-welding of high-roughness Invar alloy to sapphire using burst-mode femtosecond lasers under non-optical-contact conditions. Comparing single-pulse and burst modes, they observed that the former produced irregular interfaces with numerous cracks in sapphire, while the latter yielded smoother, more uniform interfaces with fewer cracks. Their research reduces the stringent requirements for workpiece surface flatness and clamping precision, thereby enhancing the feasibility of femtosecond laser welding for complex components and rough surfaces. The application of burst mode demonstrates precise control over temporal energy delivery, effectively managing heat accumulation and minimizing crack formation caused by thermal stress. Collectively, these studies suggest that the potential of femtosecond laser welding remains largely untapped. By innovating laser modes (e.g., single-pulse, burst) and optimizing energy management strategies, we can continuously push beyond the limitations of existing technologies. Moving forward, efforts should focus on standardizing and scaling successful laboratory welding processes while developing reliable in-line non-destructive testing methods to evaluate weld quality. Establishing correlation models between welding process monitoring techniques (e.g., plasma spectroscopy, acoustic emission) and joint strength could enable real-time assessment and closed-loop control of weld quality.

Yu et al. [131] achieved micro-welding between sapphire and copper under non-optically contacted conditions using a femtosecond laser, investigating the influence of a pre-deposited titanium thin-film interlayer on joint performance. One group of sapphire-copper joints was directly welded under the following conditions: 15 W laser power and 5 mm/s scanning speed. Another group was pretreated with a titanium nanoparticle coating on the sapphire surface using femtosecond laser-induced reverse transfer technology before undergoing the same welding process. Results indicate that direct welding formed a metallurgical bond with a shear strength of 46.4 MPa. In contrast, the coated interface developed a 0.5 μm thick titanium transition layer containing mixed nanoparticles of Ti, TiO_2_, Cu, and Al_2_O_3_, exhibiting a significantly enhanced shear strength of 115.5 MPa. Yu et al. [131] systematically compared direct welding with titanium interlayer-modified welding, conclusively demonstrating the decisive role of carefully designed nanoscale interlayers in substantially enhancing the bond strength between sapphire and high-thermal-conductivity metals (e.g., copper). The approximately 2.5-fold increase in strength represents an exceptionally significant improvement, underscoring the critical importance of optimizing the interfacial reaction layer. The LIBT technology’s pre-deposited coating offers an innovative approach. Future research should focus on deepening the understanding of the formation mechanisms and compositional control of titanium nanotransition layers to further enhance their stability. Additionally, exploring other types of nanoscale interlayer materials, such as reactive metals like Zr and Hf or their oxides, will facilitate the evaluation of the long-term reliability and failure mechanisms of these composite joints under thermal cycling conditions.

## 7. Summary and Prospects

Femtosecond laser processing of sapphire offers numerous advantages. Compared to traditional methods, this technology achieves exceptional shape accuracy and higher processing efficiency through ultra-short pulses, extremely high peak power and nonlinear interactions. By employing ablation, hybrid processing and direct micro-machining techniques, femtosecond lasers could create complex structures such as micro-holes, grooves and capillaries with high precision and minimal damage. Additionally, femtosecond lasers could modulate sapphire’s optical properties, such as absorption, reflectance and transmittance, as well as its wettability (hydrophilic/hydrophobic characteristics) and mechanical and chemical properties. This capability expands sapphire’s applications in micro-optics, sensing and biomedical fields. Optical waveguide writing and grating fabrication enable the production of low-loss waveguides, beam splitters and various grating structures spanning infrared to visible wavelengths, establishing a reliable platform for integrated photonics and high-temperature sensing. Furthermore, femtosecond lasers achieve high-strength, hermetically sealed connections between sapphire and multiple metals, effectively addressing challenges in bonding heterogeneous material interfaces. Femtosecond laser processing technology demonstrates irreplaceable advantages in high precision, minimal heat-affected zones, and true 3D machining, establishing itself as the core technology for sapphire micro/nano fabrication and functional modification.

Although femtosecond laser processing of sapphire has made some progress, it still faces challenges such as processing efficiency, cost control and process standardization. Future research should focus on the following areas:

(1) Currently, femtosecond laser processing of complex three-dimensional structures suffers from low efficiency. Future developments should focus on creating high-repetition-rate femtosecond lasers operating at megahertz frequencies with high average power, while exploring novel material interaction mechanisms to enhance material removal rates without compromising processing quality. Utilizing diffractive optical elements or spatial light modulators to split a single laser beam into multiple parallel processing beams enables simultaneous, synchronized machining of large-area sapphire substrates. This approach represents a key pathway to overcoming industrialization bottlenecks. When combined with more precise galvanometer systems and high-speed motion platforms, optimizing scanning strategies and path planning reduces idle time, thereby achieving efficient, large-area processing coverage.
(2) By leveraging machine learning and artificial intelligence algorithms to perform deep learning on vast amounts of processing parameters and results, a mapping model between laser parameters and processing quality is established. This approach rapidly derives optimal processing solutions, replacing the traditional trial-and-error method and significantly shortening the process development cycle. By integrating real-time monitoring technologies such as plasma spectroscopy and optical coherence tomography, the system dynamically tracks ablation states, defect formation, and structural morphology during processing. It employs feedback control to automatically adjust laser parameters, ensuring consistency and reliability. Additionally, a high-precision multiphysics model of femtosecond laser–sapphire interactions is developed to simulate and predict processing outcomes in virtual space, providing theoretical tools to guide actual processing, predict defects, and suppress them.
(3) On a single sapphire wafer, femtosecond laser-integrated fabrication is used to create waveguides, beam splitters, gratings, and microfluidic channels, enabling the development of “lab-on-a-chip” sensors and miniature spectrometers suitable for extreme environments. By combining surface modification, internal 3D processing, and welding techniques, this approach allows the simultaneous fabrication of macroscopic structures, micron-scale fluid channels, and nanoscale optical features within a single sapphire component. This results in the creation of superior optical windows, corrosion-resistant microreactors, and other advanced devices. Additionally, the potential of waveguides and color centers fabricated in sapphire via femtosecond lasers is being explored for quantum bit storage and transmission, advancing applications in quantum computing and quantum sensing.
(4) Investigate the processing and modification behavior of femtosecond lasers at interfaces between sapphire and other high-performance materials, such as gallium nitride and graphene, to achieve functional connections and structural fabrication of heterostructures, thereby expanding their applications in wide-bandgap semiconductor devices. Conduct in-depth studies on novel physical phenomena arising from interactions between extreme-parameter lasers, such as attosecond pulses and mid-infrared femtosecond lasers and sapphire. Explore emerging effects, including laser-induced crystalline phase transitions and amorphization control, to establish a physical foundation for developing innovative optoelectronic devices.

## Data Availability

No new data were created or analyzed in this study. Data sharing is not applicable to this article.
